# “GENYAL” Study to Childhood Obesity Prevention: Methodology and Preliminary Results

**DOI:** 10.3389/fnut.2022.777384

**Published:** 2022-03-08

**Authors:** Helena Marcos-Pasero, Elena Aguilar-Aguilar, Rocío de la Iglesia, Isabel Espinosa-Salinas, Susana Molina, Gonzalo Colmenarejo, J. Alfredo Martínez, Ana Ramírez de Molina, Guillermo Reglero, Viviana Loria-Kohen

**Affiliations:** ^1^Nutrition and Clinical Trials Unit, GENYAL Platform, IMDEA-Food Institute, CEI UAM + CSIC, Madrid, Spain; ^2^Faculty of Health Sciences, Valencian International University (VIU), Valencia, Spain; ^3^Departamento de Ciencias Farmaceúticas y de la Salud, Facultad de Farmacia, Universidad San Pablo-CEU, CEU Universities, Madrid, Spain; ^4^Nutritional Genomics and Health Unit, GENYAL Platform, IMDEA-Food Institute, CEI UAM + CSIC, Madrid, Spain; ^5^GenyalLab, GENYAL Platform, IMDEA-Food Institute, CEI UAM + CSIC, Madrid, Spain; ^6^Biostatistics and Bioinformatics Unit, IMDEA-Food Institute, CEI UAM + CSIC, Madrid, Spain; ^7^Precision Nutrition and Cardiometabolic Health, IMDEA-Food Institute, CEI UAM + CSIC, Madrid, Spain; ^8^IdisNA, Navarra Institute for Health Research, Pamplona, Spain; ^9^Center of Biomedical Research in Physiopathology of Obesity and Nutrition, Institute of Health Carlos III, Madrid, Spain; ^10^Molecular Oncology and Nutritional Genomics of Cancer, IMDEA-Food Institute, CEI UAM + CSIC, Madrid, Spain; ^11^Production and Development of Foods for Health, IMDEA-Food Institute, CEI UAM + CSIC, Madrid, Spain; ^12^Department of Production and Characterization of Novel Foods, Institute of Food Science Research (CIAL), CEI UAM+CSIC, Madrid, Spain; ^13^Departamento de Nutrición y Ciencia de los Alimentos, Facultad de Farmacia, Universidad Complutense de Madrid, Grupo de Investigación VALORNUT-UCM, Madrid, Spain

**Keywords:** pediatric obesity, early intervention, single nucleotide polymorphisms, machine learning, nutrition

## Abstract

**Objective:**

This article describes the methodology and summarizes some preliminary results of the GENYAL study aiming to design and validate a predictive model, considering both environmental and genetic factors, that identifies children who would benefit most from actions aimed at reducing the risk of obesity and its complications.

**Design:**

The study is a cluster randomized clinical trial with 5-year follow-up. The initial evaluation was carried out in 2017. The schools were randomly split into intervention (nutritional education) and control schools. Anthropometric measurements, social and health as well as dietary and physical activity data of schoolchildren and their families are annually collected. A total of 26 single nucleotide polymorphisms (SNPs) were assessed. Machine Learning models are being designed to predict obesity phenotypes after the 5-year follow-up.

**Settings:**

Six schools in Madrid.

**Participants:**

A total of 221 schoolchildren (6–8 years old).

**Results:**

Collected results show that the prevalence of excess weight was 19.0, 25.4, and 32.2% (according to World Health Organization, International Obesity Task Force and Orbegozo Foundation criteria, respectively). Associations between the nutritional state of children with mother BMI [β = 0.21 (0.13–0.3), *p* (adjusted) <0.001], geographical location of the school [OR = 2.74 (1.24–6.22), *p* (adjusted) = 0.06], dairy servings per day [OR = 0.48 (0.29–0.75), *p* (adjusted) = 0.05] and 8 SNPs [rs1260326, rs780094, rs10913469, rs328, rs7647305, rs3101336, rs2568958, rs925946; *p* (not adjusted) <0.05] were found.

**Conclusions:**

These baseline data support the evidence that environmental and genetic factors play a role in the development of childhood obesity. After 5-year follow-up, the GENYAL study pretends to validate the predictive model as a new strategy to fight against obesity.

**Clinical Trial Registration:**

This study has been registered in ClinicalTrials.gov with the identifier NCT03419520, https://clinicaltrials.gov/ct2/show/NCT03419520.

## Introduction

Obesity is a complex, chronic and multifactorial disease, originated as an interaction between genetic and environmental factors (1). The prevalence of overweight and obese children is rising every year. Specifically, in compliance with the WHO, the number of overweight and obese children aged 0–5 years increased from 32 million globally in 1990 to 41 million in 2016. And it is expected to increase to 70 million by 2025 if these trends continue (2). The situation in Spain is also alarming, with a prevalence of 23.2% of overweight (22.4% boys and 23.9% girls), and 18.1% of obesity (20.4% boys and 15.8% girls) according to data from the ALADINO study carried out by the Spanish Agency for Consumer Affairs, Food Safety and Nutrition (3).

Childhood obesity usually leads to adulthood obesity, which increases the risk of developing certain diseases, such as hypertension, type 2 diabetes and cardiovascular diseases, prematurely (4–6). This early age has been identified as a key point for the implementation of healthy dietary and lifestyle patterns. Thus, the home and schools provide a useful environment to develop educational and lifestyle interventions for school-age children (7).

There is no doubt about the multifactorial etiology of obesity in which socio-cultural, dietetic, environmental and genetic factors are involved (8–11). However, current knowledge is still insufficient to determine the relative importance of these different factors, having a complex network of associations between them (12). In this regard, machine learning techniques represent a powerful prediction tool through their great ability to big data analysis. Thus, Machine learning represents a tool based on a set of algorithms that can characterize, adapt, learn, predict, and analyze data, increasing the knowledge of obesity and offering possibilities of predicting the disease with unprecedented precision. These techniques have been proposed as a potential tool to predict a future excess of body weight and its comorbidities. There are several predictive machine learning algorithms such as neural networks, decision tree analysis or random forest. Each of them should be used according to the purpose and nature of the study variables (13).

Considering all the above-mentioned aspects, the main objective of the GENYAL study is to design and validate a machine learning-based predictive model that identifies children who would benefit most from actions aimed at reducing the risk of obesity and its complications, considering both environmental and genetic factors, and applicable at the beginning of the school stage. The nutritional education developed in the intervention's schools will be also evaluated as part of the predictive model. This article describes the methods and analyses that will be applied. In addition, it summarizes some preliminary results obtained after the first year of the data collection.

## Methods

### Type of Study and Duration

**Figure 1:**
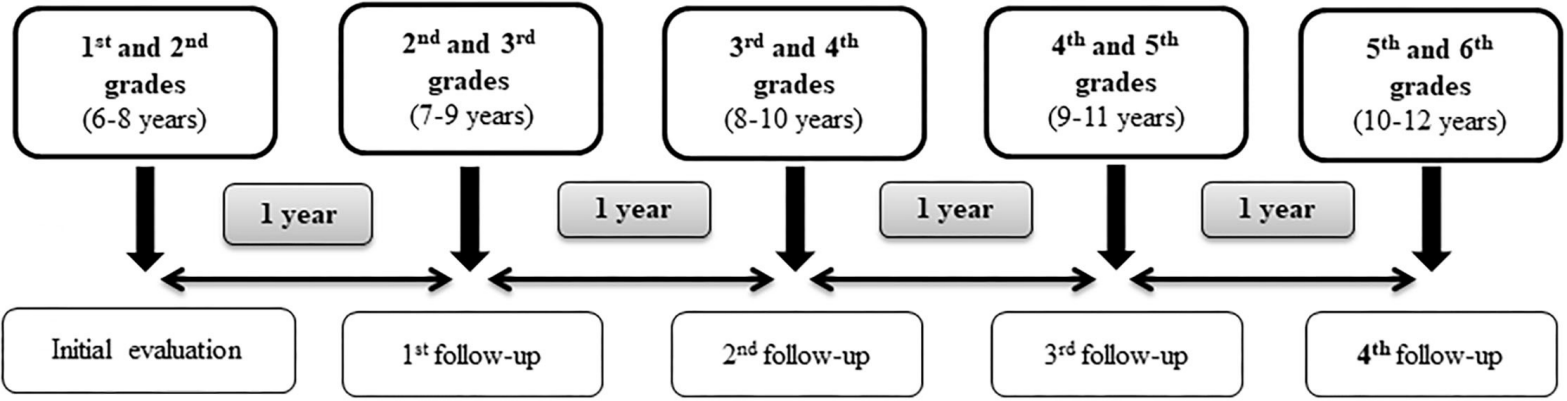
Timetable of the study.

The present study is a cluster randomized clinical trial with 5-year follow-up intervention based on nutritional education, annual anthropometric measurement evaluations and data collection from questionnaires. Saliva samples were collected for all the schoolchildren in the initial evaluation (2017) in order to obtain genetic information. The final evaluation will be carried out 4 years after the initial intervention, which corresponds to the end of the primary school (Figure 1). The study is therefore expected to last 5 years, from 2016 to 2017 academic year to 2021–2022. Table 1 provides a schedule of activities and interventions throughout the study.

**Table 1:** GENYAL study timeline.

	**Study period in years**						
	**Enrolment (T-1)**	**Allocation (T0)**	**T1**	**T2**	**T3**	**T4**	**T5**
**Enrolment**							
Study approval	x						
Schools recruitment	x						
Informed consent	x						
Group allocation		x					
**Assessments**							
Sending of questionnaires			x	x	x	x	x
Anthropometric measurements			x	x	x	x	x
Saliva samples collection			x				
Questionnaires collection			x	x	x	x	x
Machine learning model results							x
**Intervention group**							
Sending educational material for parents, teachers, and schoolchildren			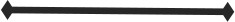				
Nutrition education workshops and talks			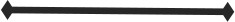				

*T-1 (enrolment) and T0 = 2016/2017; T1 = 2017/2018; T2 = 2018/2019; T3 = 2019/2020; T4 = 2021/2021; T5 = 2021/2022*.

### Recruitment, Sample Size, and Sample Characteristics

Due to the nature of the study as a clinical trial, the large number of variables necessary for the design of the preventive model with machine learning (each of them of a very different nature), and the duration of 5 years, a statistically robust sample size could not be implemented. Furthermore, the *Consejer*í*a de Educación e Investigación de la Comunidad de Madrid* was responsible for the selection of six representative schools of the Autonomous Community of Madrid (ACM) (Spain) (two in the north, two in the center and two in the city's south zone), considering the number of students per center and the average socioeconomic level of the districts and neighborhoods. Therefore, the selection was representative of the average income of the ACM households (14). All the School Boards approved the participation in the study and included a total of 569 potential children participants from different districts of Madrid: Chamberí, Hortaleza, Carabanchel, Puente de Vallecas, and Moncloa-Aravaca.

### Inclusion and Exclusion Criteria

The inclusion criteria to participate in the study were: being in 1st or 2nd grade of primary school and having an informed consent signed by at least one of the parents.

Exclusion criteria were not attending school during the evaluation days or having planned not to stay at the school the following years.

### Randomization

In order to avoid cross-contamination between intervention and control subjects, randomization was carried out by school center instead of individually. Thus, participating schools were randomly and proportionally stratified into two groups: intervention schools and control schools, considering the number of participants per center, their geographic area and their socioeconomic status.

The randomization procedure was carried out with the statistical software R version 3.4 (www.r-project.org).

### Ethical Aspects and Data Processing

Protocols and methodology used in the present study comply with the ethical principles for research involving human subjects laid down in the Declaration of Helsinki (1964) and its modifications. The study was approved by the Research Ethics Committee of the IMDEA Food Foundation (PI:IM024; Approval date: March 29th, 2016) and it has been registered in ClinicalTrials.gov with the identifier NCT03419520. School centers and families were informed in detail about the different stages of the project both, orally and in writing. Signed informed consent from at least one of the parents were collected by the researchers prior to the first evaluation. This document included a specific consent to DNA extraction and the evaluation of polymorphisms from the saliva samples. In addition, it included a section on the storage of the remaining samples as a collection registered, according to Spanish legislation (Royal Decree 1716/2011, of November 18th).

Data compiled along the study are going to be processed using a web application that applies dissociation criteria making the volunteers' data anonymous, in compliance with the current Spanish legislation (Organic Law 15/1999 of December 13th, on the protection of Personal Data) and may be used for scientific purpose as publications and conferences. Only the researchers directly related to the study will be allowed to access data.

### Selection of Single Nucleotide Polymorphisms

A total of 26 single nucleotide polymorphisms (SNPs) associated with a higher risk of early-age onset of obesity and its comorbidities were selected. The selection was made considering the biological activity of each SNP, Caucasian allele frequencies and the scientific evidence that supports the association between the presence of the polymorphism and the risk of developing overweight, obesity or its complications. The sum of the risk alleles will further be used to design a genetic risk score.

Different databases such as 1,000 Genomes, HapMap, Pubmed, GWAS Central, GWAS Catalog or Ensembl were used. Table 2 shows the selected 26 SNPs, which will be included in the predictive model.

**Table 2:** Single nucleotide polymorphisms selection.

**Gene^a^**	**Name**	**Function**	**Location^b^**	**Alleles**		**WT**	**MAF^b^**	**Frequency^b^**	**Association**	**References**
*APOA5*	rs662799	Upstream gene variant	Chromosome 11:116792991	G	A	A	0.16 (G)	G: 0.076/A: 0.924 G|G: 0.010; A|A: 0.859; A|G: 0.131	Overweight and obesity, lipid profile, CVD, MS	(15–19)
*BDNF*	rs925946	Intron variant	Chromosome 11:27645655	T	G	G	0.25 (T)	T: 0.338/G: 0.662 T|T: 0.141; G|T: 0.394; G|G: 0.465	Overweight and obesity, calcium consumption, MS, waist/hip index, abdominal obesity	(20–25)
*ETV5*	rs7647305	Intron variant	Chromosome 3:186116501	T	C	T	0.24 (T)	T: 0.227/ C: 0.773 T|T: 0.101; C|T: 0.253; C|C: 0.646	Overweight and obesity, early menarche, BP	(20, 24, 26, 27)
*FTO*	rs3751812	Intron variant	Chromosome 16:53784548	G	T	G	0.22 (T)	G: 0.556/T: 0.444 G|G: 0.303; G|T: 0.505; T|T: 0.192	Overweight and obesity, lipid profile	(28–31)
*FTO*	rs7190492	Intron variant	Chromosome 16:53794840	A	G	G	0.30 (A)	A: 0.343/G: 0.657 A|A: 0.131; A|G: 0.424; G|G: 0.444	Overweight and obesity	(28, 29, 32)
*FTO*	rs8050136	Intron variant	Chromosome 16:53782363	C	A	A	0.32 (A)	C: 0.556/A: 0.444 C|C: 0.303; A|C: 0.505; A|A: 0.192	Overweight and obesity, T2DM	(28, 29, 33–36)
*FTO*	rs9939609	Intron variant	Chromosome 16:53786615	T	A	A	0.34 (A)	T: 0.556/A: 0.444 T|T: 0.303; A|T: 0.505; A|A: 0.192	Overweight and obesity, extreme childhood obesity, T2DM, MS, early menarche, fat mass	(20, 27, 37–41)
*GCKR*	rs1260326	Missense variant	Chromosome 2:27508073	T	C	C	0.29 (T)	T: 0.429/C: 0.571 T|T: 0.172; C|T: 0.515; C|C: 0.313	Overweight and obesity, MS, lipid profile, glycemic profile	(42–45)
*GCKR*	rs780094 a	Intron variant	Chromosome 2:27518370	T	C	C	0.30 (T)	T: 0.409/C: 0.591 T|T: 0.162; C|T: 0.495; C|C: 0.343	Overweight and obesity, lipid profile, MS, glycemic profile, fatty liver, T2DM	(42, 43, 46–50)
*GNPDA2*	rs10938397	Intergenic variant	Chromosome 4:45180510	A	G	A	0.33 (G)	A: 0.576/G: 0.424 A|A: 0.333; A|G: 0.485; G|G: 0.182	Overweight and obesity, extreme childhood obesity, early menarche, T2DM, glycemic profile, fat mass, waist circumference, waist-height ratio, central obesity	(20, 27, 40, 41, 51)
*KCTD15*	rs368794	Intergenic variant	Chromosome 19:33829547	T	A	A	0.42 (T)	T: 0.298/A: 0.702 T|T: 0.081; A|T: 0.434; A|A: 0.485	Overweight and obesity, fat mass	(20, 41, 52, 53)
*LEPR*	rs1137101	Missense variant	Chromosome 1:65592830	A	G	A	0.42 (A)	A: 0.515/G: 0.485 A|A: 0.253; A|G: 0.525; G|G: 0.222	Obesity, lipid profile, glycemic profile, caloric intake, T2DM	(54–58)
*LPL*	rs328	Stop gained	Chromosome 8:19962213	C	G	C	0.09 (G)	C: 0.874/G: 0.126 C|C: 0.768; C|G: 0.212; G|G: 0.020	Lipid profile, CVD	(59–63)
*MC4R*	rs17782313	Intergenic variant	Chromosome 18:60183864	T	C	T	0.24 (C)	T: 0.742/C: 0.258 T|T: 0.515; C|T: 0.455; C|C: 0.030	Overweight and obesity, extreme childhood obesity, MS, T2DM, abdominal obesity, fat mass, waist circumference, waist-height ratio	(22, 41, 64–69)
*NEGR1*	rs2568958	Upstream gene variant	Chromosome 1:72299433	G	A	G	0.32 (G)	G: 0.364/A: 0.636 G|G: 0.141; A|G: 0.444; A|A: 0.414	Overweight and obesity, extreme childhood obesity, fat mass.	(20, 41, 70)
*NEGR1*	rs3101336	Upstream gene variant	Chromosome 1:72285502	T	C	T	0.32 (T)	T: 0.364/C: 0.636 T|T: 0.141; C|T: 0.444; C|C: 0.414	Overweight and obesity, severe early-onset obesity.	(70–72)
*NPY*	rs16147	Upstream gene variant	Chromosome 7:24283791	T	C	T	0.48 (T)	T: 0.485/C: 0.515 T|T: 0.273; C|T: 0.424; C|C: 0.303	Overweight and obesity	(73–75)
*PPARγ*	rs1801282	Missense variant	Chromosome 3:12351626	C	G	C	0.07 (G)	C: 0.904/G: 0.096 C|C: 0.828; C|G: 0.152; G|G: 0.020	Overweight and obesity, glycemic profile, CVD, T2DM, Skin folds	(76–82)
*SEC16B*	rs10913469	Intron variant	Chromosome 1:177944384	T	C	C	0.23 (C)	T: 0.758/C: 0.242 T|T: 0.576; C|T: 0.364; C|C: 0.061	Overweight and obesity, waist circumference, fat mass, early menarche	(27, 28, 30, 55, 83, 84)
*TCF7L2*	rs7903146	Intron variant	Chromosome 10:112998590	C	T	T	0.23 (T)	C: 0.687/T: 0.313 C|C: 0.525; C|T: 0.323; T|T: 0.152	IMC, T2DM, MS, glycemic profile, height, lipid profile in DM patients	(85–93)
*TMEM18*	rs2867125	Intergenic variant	Chromosome 2:622827	T	C	C	0.14 (T)	T: 0.162/C: 0.838 T|T: 0.051; C|T: 0.222; C|C: 0.727	Overweight and obesity	(30, 94, 95)
*TMEM18*	rs4854344	Intergenic variant	Chromosome 2:638144	G	T	G	0.15 (G)	G: 0.167/T: 0.833 G|G: 0.051; G|T: 0.232; T|T: 0.717	Overweight and obesity, waist circumference, visceral fat and SBP	(83, 96–98)
*TMEM18*	rs6548238	Regulatory region variant	Chromosome 2:634905	T	C	C	0.12 (T)	T: 0.167/C: 0.833 T|T: 0.051; C|T: 0.232; C|C: 0.717	Overweight and obesity, extreme childhood obesity, fat mass, early menarche, waist circumference,	(20, 27, 41, 55, 65, 97, 99, 100)
*TMEM18*	rs7561317	Intergenic variant	Chromosome 2:644953	A	G	A	0.16 (A)	A: 0.162/G: 0.838 A|A: 0.040; A|G: 0.242; G|G: 0.717	Overweight and obesity	(21, 22, 24, 33, 40, 101, 102)
*UCP2*	rs659366	Upstream gene variant	Chromosome 11:73983709	C	T	C	0.41 (T)	C: 0.636/T: 0.364 C|C: 0.414; C|T: 0.444; T|T: 0.141	Overweight and obesity, glycaemia	(103–105)
*UCP3*	rs1800849	5 prime UTR variant	Chromosome 11:74009120	G	A	G	0.19 (A)	G: 0.768/A: 0.232 G|G: 0.586; A|G: 0.364; A|A: 0.051	Overweight and obesity, glycaemia, lipid profile, waist hip ratio	(103, 106–110)

a *Approved name (111)*.

b *Ensembl (https://www.ensembl.org/index.html). Location, For forward strand*.

*WT, wild type; MAF, Minor allele frequency; Frequency, Allele and genotype; APOA5, apolipoprotein A5; BDNF, brain derived neurotrophic factor; ETV5, ETS variant 5; FTO, FTO, alpha-ketoglutarate dependent dioxygenase; GCKR, glucokinase regulator; GNPDA2, glucosamine-6-phosphate deaminase 2; KCTD15, potassium channel tetramerization domain containing 15; LEPR, leptin receptor; LPL, lipoprotein lipase; MC4R, melanocortin 4 receptor; NEGR1, neuronal growth regulator 1; NPY, neuropeptide Y; PPARγ, peroxisome proliferator activated receptor gamma; SEC16B, SEC16 homolog B, endoplasmic reticulum export factor; TCF7L2, transcription factor 7 like 2; TMEM18, transmembrane protein 18; UCP2, uncoupling protein 2; UCP3, uncoupling protein 3; CVD, cardiovascular disease; MS, metabolic syndrome; BP, blood pressure; T2DM, type 2 diabetes mellitus; SBP, systolic blood pressure*.

### Questionnaires

Different questionnaires were designed based on other surveys used in similar studies to facilitate the comparison of the results. All of them are annually sent to families by email or in the paper format according to the parents' preference and are filled by at least one of the parents. The information collected is summarized in Table 3.

**Table 3:** Content of the questionnaires used in the study.

**Questionnaire**	**Variables**
**Characteristics of parents and family**	
Social, health and demographic	People living with the schoolchildren, number of siblings, family structure, level of education, employment situation, profession, country of birth, years living in Spain, income level, diagnosis of disease, smoking habit, body weight, height and date of birth.
Diet	Daily intakes, frequency of breakfast per week, breakfast foods, reasons for stop breastfeeding, desire to extend the breastfeeding period, perception of their child's diet.
Physical activity	Distance between school and home, weekly hours of physical activities, frequency of physical activity shared with their child, town conditions for doing outdoor activities.
**Characteristics of children**	
Social, health and demographic	Country of birth, years living in Spain, adopted or foster child, diagnosis of disease, body weight and height reported by their parents and date of birth.
Diet	Diet changes over the last year and reasons, number of daily intakes, frequency of breakfast per week, place where they have breakfast, breakfast foods, place where they have lunch, frequency of eating in fast food restaurants, breastfeeding, beikost's start date.
	48-h dietary record
	KIDMED questionnaire
Physical activity	Sleeping hours, type of transport used to go to school and back home, outdoor activities, time usually spend watching TV, playing video games or using the computer, presence of TV or computer games in their bedroom, weekly hours of physical activities and children's physical activity level according to their parents
	48-h physical activity questionnaire

Regarding social, health and demographic data, parents annually complete a self-reported questionnaire that includes different personal questions based on the surveys used in the ALADINO and ELOIN studies (3, 112).

Dietary information is gathered using a 48-h food record of 2 non-consecutive days, a weekday and a weekend day, as recommended by the European Food Safety Authority guidelines (113). Afterwards, the data are tabulated and analyzed using the DIAL software (*Alce Ingenier*í*a*, Madrid, Spain) (114) in order to obtain information about macro and micronutrients.

Moreover, the adherence to the Mediterranean diet pattern is assessed using the “KIDMED Mediterranean Diet Quality Index” in addition to general questions about the dietary habits of the children and their parents. The KIDMED questionnaire consists of a total of 16 dichotomous questions that must be answered affirmatively or negatively to obtain a score (115).

Physical activity and free time data about the children and their parents are gathered using a questionnaire with different sections adapted and modified from the ALADINO (3) and the ELOIN (112) studies. In addition, a 48-h physical activity record is collected, corresponding to 24 h of a weekday and a complete weekend day (116). In the physical activity record, parents had to specify the time that their children spent during 24 h of a week day and 24 h of a weekend day doing different activities, including resting hours and activities with a variable level of intensity (very light, light, moderate and intense). The time spent doing each activity is multiplied by the corresponding activity coefficient defined by the WHO (117), added and divided by 24, obtaining the Individual Physical Activity Coefficient (IPAC). Then, the IPAC corresponding to a weekday is multiplied by 5 and the weekend IPAC by 2, both results are added and divided by 7, thus, obtaining the median physical activity per individual. Afterwards, it is necessary to convert the IPAC into a Physical Activity Coefficient (PAC) according to sex, therefore an equivalence is made between the IPAC and the PAC proposed by the Institute of Medicine (118). Finally, participants are classified into sedentary, lightly active, active and very active in line with their PAC.

All these data and information are collected every year on equal terms.

### Anthropometric and Blood Pressure Measurements

These data are collected in the school centers, early in the morning, by previously trained nutritionists, following standardized protocols and WHO international instructions for this age group (117). For the anthropometric measurements, children had to wear a T-shirt and gym shorts. All measures are taken twice, and the average is used for the analyses.

Height is determined using a Leicester height rod with an accuracy of 1 mm (Biological Medical Technology SL, Barcelona, Spain). Body weight and fat mass percentage are assessed using a BF511 Body Composition Monitor (BF511- OMRON HEALTHCARE UK, LT, Kyoto, Japan). Furthermore, fat mass percentage is classified according to the tables offered by OMROM Healthcare (119). Waist and brachial circumferences measurements are taken using a non-elastic tape (KaWe Kirchner & Wilhelm GmbH, Asperg, Germany; range 0–150 cm, 1 mm of precision). The waist circumference measurements obtained are classified by percentiles in compliance with Fernández et al. (120). Triceps skinfolds are taken following the International Society for the Advancement of Kinanthropometry guidelines (121) using a mechanic caliper (HOLTAIN LTD. CRYMYCH UK 10 g/mm^2^ constant pressure; range 0–39 mm and 0.1 mm of precision) and the results obtained are ranked according to percentiles proposed by Frisancho AR (122).

Using these data, other variables of interest are calculated. In particular, BMI is calculated as the body weight divided by the squared height (kg/m^2^). There is not a universal technique to classify the BMI values in the pediatric collective, therefore, the results are ranked according to the percentiles of Faustino Orbegozo Eizaguirre Foundation, reviewed in 2011 (120), International Obesity Task Force reviewed in 2000 (123), and WHO reviewed in 2007 (124). The results of overweight and obesity rates are unified as a single category called excess weight (EW). The arm muscular and fat areas are obtained using the equations proposed by Mataix Verdú and López Jurado (125) and López-Sobaler and Quintas Herrero (126), respectively. The protein and caloric reserves are calculated by Frisancho AR equations (122). Waist/height ratio is calculated as waist circumference (cm)/height (cm) and it was classified according to Panjikkaran et al. and Ashwell investigations (127, 128). Height/age index is rated in percentiles according to Fernández et al. (120).

For blood pressure monitoring, an automatic digital monitor is used (OMRON M3-Intellisense) using a cuff suitable for children. The results are classified according to the percentiles established by the Spanish Association of Pediatrics (129).

All these measurements are repeated every year on equal terms.

### Compiling Saliva Samples, DNA Extraction and Genotyping

Buccal smears were collected for DNA extraction following standardized protocols. For this purpose, a sterile swab free of human RNAse, DNAse and DNA (300263DNA-Hisopos Deltalab polystyrene and polyester) was used. Children had to have their mouth clean and avoid eating or drinking 30 min prior to collection. Three samples were taken per children, each one identified with the number corresponding to the order of extraction, to ensure traceability. As the samples were collected, they were directly stored in refrigeration until all the children were evaluated. Immediately after, they were frozen at −80°C until their processing.

Genomic DNA was extracted from the buccal swabs using the INVISORB® SPIN TISSUE MINI KIT (Stratec), according to the manufacturer's instructions. Samples were lysed in the presence of proteinase K and a specific lysis buffer. The lysate was then purified and finally, it was eluted in a free EDTA solution.

For genotyping, the DNA samples were loaded in TaqMan® OpenArray® Real-Time PCR plates (Life Technologies Inc., Carlsbad, CA) already configured with the specific selected SNPs with specific waves for each allele, marked with a different fluorophore to determine the genotype. This process was made using the OpenArray® AccuFill™ System (Life Technologies Inc., Carlsbad, CA). Once it was charged, a PCR performed and the chips were read in the QuantStudio® 12K Flex Real-Time PCR Instrument (Life Technologies Inc., Carlsbad, CA). Results were analyzed using the TaqMan® Genotyper software (Life Technologies Inc., Carlsbad, CA), which automatically assigns the genotype to each sample according to the amount of detected signal for each fluorophore.

The duplicate analysis was used to validate the genotyping result.

### Design of Educational Tools and Implementation of the Nutritional Education Programme

For the implementation of the nutritional education programme in the “intervention schools”, three different kinds of guides were designed aimed at parents, children and teachers. All this information was developed and adapted to the participants' age by the nutritionists from the IMDEA Food Foundation. This material is sent to parents and educational centers in different modules adapted to parents, students, and teachers. The same modules include different activities and topics each year according to the children's growth. The sending strategy follows a protocol, and it will be maintained until the end of the study, through email or regular delivery, as the receiver may prefer.

Moreover, some workshops are being carried out and are summarized in Table 4.

**Table 4:** Educational components of GENYAL study.

	**Format**	**Module**	**Objective**
Children	Notebook activities	Food and nutrients	Essential nutrients: definition, function and sources
		The food pyramid	To identify the different food categories and explore which foods are in each group
	Workshops	The food pyramid	To draw a food pyramid and to create healthy dishes with the different food categories
Parents	Nutritional guide	Introduction	To know the bases and objectives of the study
		Food and nutrients	Essential nutrients: definition, function and sources
		The food pyramid	To identify the different food categories and which foods are in each group
	Workshops	Preliminary results	To know about the preliminary results of the study and the recommendations derived from them
Teachers	Nutritional guide	Introduction	To know the bases and objectives of the study
		Food and nutrients	Essential nutrients: definition, function and sources
		The food pyramid	To identify the different food categories and which foods are in each group
	Workshops	Preliminary results	To know about the preliminary results of the study and the recommendations derived from them

The validation of this tool is expected to be carried out through the impact generated over the years of the study, measured as the evolution of anthropometric variables and the dietary habits, between control and intervention schools. Moreover, parents and teachers along the study will evaluate all the material.

### Statistical Analysis

Descriptive analyses of the baseline data were performed by computing for the categorical variables the class's absolute and relative frequencies, and for the quantitative variables the mean, median, standard deviation, interquartile range, maximum and minimum. To check the homogeneity of the two groups in the case of quantitative variables, *t*-tests were used for normally-distributed variables, or Mann-Whitney *U*-test as non-parametric alternative. In the case of categorical variables, Chi-Square or Fischer exact tests were used. The association between anthropometric and dietary, social, health and SNP variables were performed by linear or logistic regressions. The Bonferroni correction was applied for multiple tests. In addition, for the SNPs variables, the Hardy-Weinberg equilibrium condition was tested by means of Chi-Square tests. All analyses were conducted with R Statistical Software version 3.41. Statistical tests used a 0.05 significance level, in two-tailed tests.

Regarding Machine Learning models, they will be derived to predict the BMI from all the analyzed variables *after the 5-year follow-up*. Both classification (after dichotomization of the BMI) and regression models will be considered, and Random Forest will be applied. It has been observed that the use of Random Forest improves the predictive model's performance, creating a more effective predictive model than the one that could be obtained using decision tree or logistic regression techniques (13). The predictive power of the models will be evaluated and internal cross-validation and external validations with external datasets will be implemented. Variable importance analyses will be performed in order to quantify the relative weights of the different variables in the prediction of BMI. The model will be iteratively improved by refitting with new data along with the successive yearly evaluations during the study.

## Results

**Figure 2:**
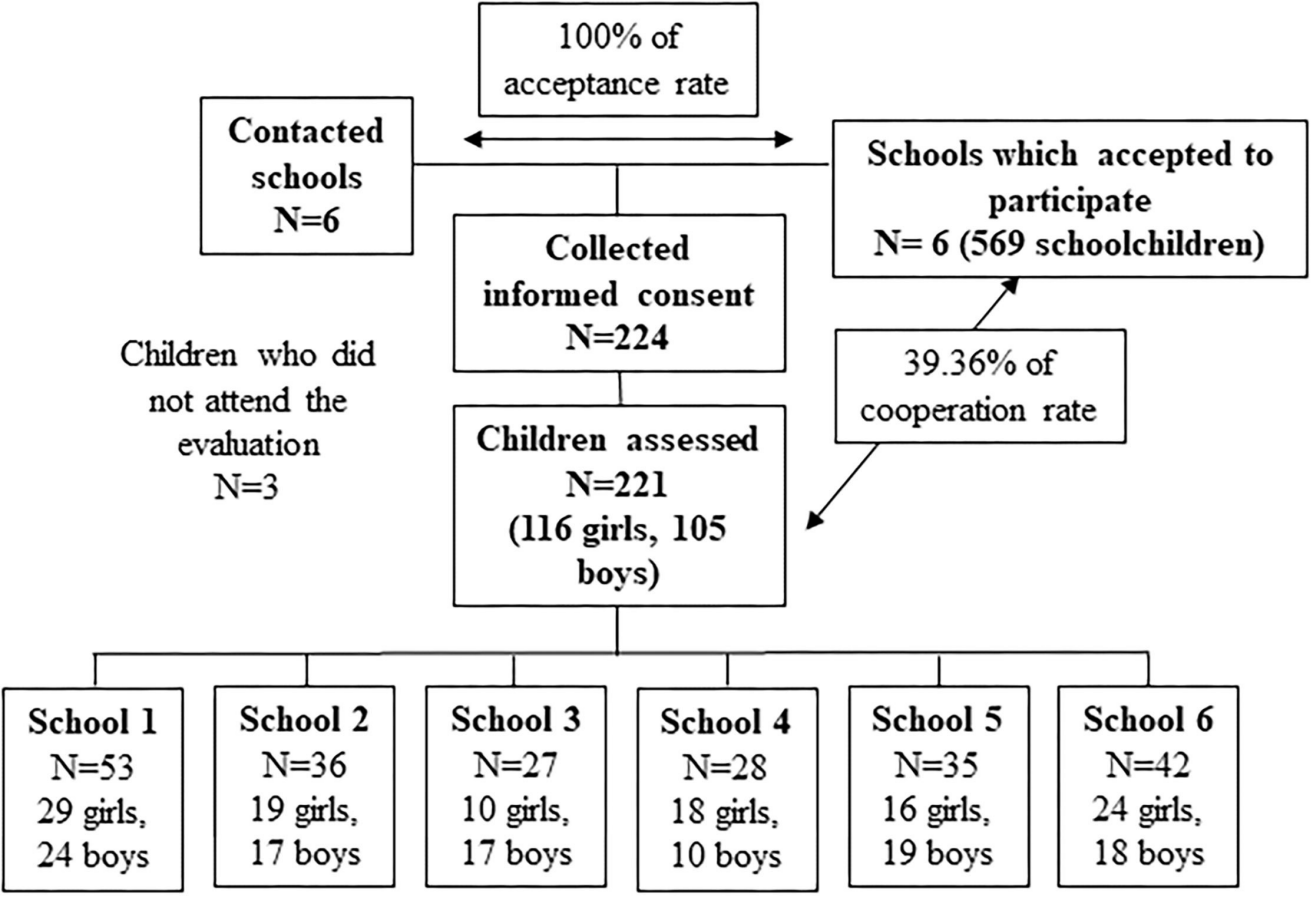
Volunteers flow chart and final sample.

Parents of 224 children (116 girls and 105 boys) accepted to participate in the study and signed the informed consent. It shows a collaboration rate of 39%. Finally, 221 children were evaluated, since three did not attend the initial evaluation (Figure 2).

Among the total number of students enrolled in the study, 115 belonged to intervention schools while 106 were in control schools. Tables 5, 6 show the basal main characteristics of the study sample according to the corresponding control or intervention group.

**Table 5:** Main socio-demographic and anthropometric characteristics of the schoolchildren by groups (intervention schools vs. control schools).

	**Intervention group** **(*n* = 115)**	**Control group** **(*n* = 106)**	***p***
**Socio-Demographic**			
**Spanish (%)**			
– No	1.90	3.00	0.678
– Yes	98.10	97.00	
**Spanish mother (%)**			
– No	35.00	23.50	0.074
– Yes	65.00	76.50	
**Anthropometry**			
Age (years)	6.73 ± 0.76	6.77 ± 0.69	0.505
**Sex (%)**			
– Girls	52.20	42.50	0.148
– Boys	47.80	57.50	
Fat mass (%)	21.23 ± 7.56	19.84 ± 6.71	0.159
Muscle mass (%)	27.97 ± 3.00	28.01 ± 2.97	0.924
BMI (kg/m^2^)	17.21 ± 2.99	16.68 ± 2.24	0.319

**Table 6:** Main dietary and physical activity characteristics of the schoolchildren by groups (intervention schools vs. control schools).

	**Intervention group** **(*n* = 115)**	**Control group** **(*n* = 106)**	***p***
**Physical activity**			
Physical activity rate	1.61 ± 0.11	1.55 ± 0.09	<0.001
Number of hours dedicated to moderate and vigorous activities	3.53 ± 1.72	3.96 ± 1.89	0.068
**Diet**			
Total caloric value (kilojoules):	7,695.97 ± 1,962.52	7,678.59 ± 1,463.50	0.378
– Carbohydrates %	45.00 ± 5.15	43.94 ± 5.43	0.154
– Lipids %	38.40 ± 4.91	39.52 ± 5.09	0.116
– Proteins %	16.57 ± 2.25	16.52 ± 2.09	0.971
Total fiber (g)	18.54 ± 6.37	17.78 ± 5.19	0.481
KIDMED score	6.33 ± 1.98	6.67 ± 1.82	0.251

According to the preliminary results obtained after the first year of the evaluation, 32.2% of the students presented EW, taking into account the WHO criteria. These figures were higher when IOFT standard (25.4%) or the national criteria of the Orbegozo Foundation (19.0%) were applied.

A significative association between the nutritional state of children and mother BMI [β = 0.21 (0.13–0.3), *p* (adj) <0.001], geographical location of the school [OR = 2.74 (1.24–6.22), *p* (adj) = 0.06], dairy servings per day [OR = 0.48 (0.29–0.75), *p* (adj) = 0.05] and eight of the total SNPs studied [rs1260326, rs780094, rs10913469, rs328, rs7647305, rs3101336, rs2568958, rs925946; *p* (not adj) <0.05] were found (data adjusted for sex and age).

## Discussion

The GENYAL study to prevent child obesity is, to our knowledge, the first interventional trial in Spanish schoolchildren aiming to provide preventive and therapeutic approach based on a high degree of evidence for early obesity through machine learning.

The baseline data and the associations observed after the first analysis support the evidence that environmental and genetic factors play a role in the development of childhood obesity.

According with the results, for each point in the BMI of the mother, the BMI of the child was increased 0.21 kg/m^2^. It shows that among the multiple risk factors for the development of obesity in children, parental obesity is one of the most impactful as a result of both genetic and environmental interactions. Children imitate their parents, therefore, the parents' dietary habits and PA are more likely to be reproduced by their descendants (130).

With reference to the location of the school, that represent the socioeconomic level, presented a close relationship with the presence of EW, with a decreasing distribution of risk from south to north area. These results are consistent with other studies that have shown how the socioeconomic status of the school correlates with the prevalence of overweight and obesity as it increases the likelihood that schoolchildren will follow a diet rich in energy-dense, low-cost foods, as well as fewer opportunities to practice sport (131).

Regarding dietary aspects, dairy servings per day showed a protective effect against EW. It could be related to several factors such as if this food is a source of calcium, peptides, bioactive compounds, etc. They have been studied due to their relationship in the appetite control and other mechanisms involved in controlling weight (132, 133). These results highlight the important role that this group of foods could have for the prevention of weight overload.

The study of these factors in the child population and the social context of Madrid is minimal, having found a single study with similar sociodemographic characteristics (134). Nevertheless, no intervention was performed in this study, nor was genetic data collection, thus the GENYAL project is shown as a novel study in this regard.

In this study, a significant association (data adjusted for sex and age) between the nutritional status of schoolchildren and 8 SNPs was found (rs1260326, rs780094, rs10913469, rs328, rs7647305, rs3101336, rs2568958, rs925946). In previous studies, these polymorphisms have been associated with adiposity traits and their related comorbidities (Table 2). Genetic factors play an essential role in the development of obesity (135). Thus, knowing which ones are associated with excess weight early in life could contribute to obesity early detection.

Conversely, it is important to note that current knowledge is insufficient to determine the relative importance of these different factors. Therefore, new techniques are needed to be used as predictive instruments (12). Currently, machine learning is considered an extremely valuable tool in the medical field, since it is capable of providing diagnostic and early detection strategies for diseases through the analysis of large datasets (136). Prevention plays a crucial role in controlling the high obesity prevalence, so machine learning techniques have already been used for the prediction of the BMI in children (137). However, the current predictive model would be the first to include obesity-related SNPs as genetic information, as well as anthropometric, social and lifestyle variables.

Regarding the last results from the Commission on Ending Childhood Obesity, the implementation of integral programs that promote healthy environment in schools is recommended with the objective of ensuring that children grow well and develop healthy habits (138). Nevertheless, although Spain is one of the countries where more intervention studies to prevent obesity have been developed (139), the politic strategies to prevent chronic illnesses such as overweight and obesity are not defined, and even show very low evidence of efficiency, according to the last data revised by the Cochrane Database (140).

According to the latest scientific research, the intervention studies in schools which include family and community spheres, implementing actions to promote healthy food and physical activity, are the most effective (5, 46). This study has been designed to elaborate strategies and to work as a multidisciplinary team reinforcing the educational sphere of the participating children and their environment, school, and family.

One of the strong points of our study consists in the implementation, and subsequent validation, of educational tools for students, their parents and teachers, applying a nutritional education method that promotes healthy dietetic habits and physical exercise, both in schools and outside. The importance of the validation of these educational strategies lay on a large number of studies with contradictory results, which might be partly explained by the fact that many researches may have lacked statistical power to detect changes in the results of interest related to adiposity or children's nutritional status (5). In the present study, the educational approaches to the intervention schools will be held for 5 years, in line with the annual anthropometric assessments. Their utility will be evaluated taking into account the evolution of the anthropometric and dietetic results annually collected. Moreover, the presence or absence of the educational program (control and intervention schools) will be included as an input dichotomous variable in the predictive model, evaluating its influence on the predicted BMI. This will allow us to detect differences in the body composition between intervention and control schools, enabling us to assess the impact of educational support.

Another strength of the present study is the selection of 26 SNPs for early prevention of obesity, by making an extensive bibliographic research, as shown in Table 2. The nutritional genomic tools would be very useful in the research and prevention of obesity, and they would be an important support in public health applications. Obesity is a multifactorial illness, where the genetic variants involved are dispersed along the whole genome. Although SNPs have been cataloged as the best indicators to predict obesity risk (141), several studies suggest that much remains to be discovered. There is a lot of interest in predicting the appearance of chronic diseases at an early age (142). According to the last review about precision nutrition (143), the creation of a genetic risk score may let us determine the risk of developing obesity or other chronic pathologies related to the individual genetic component, and even be able to predict the expected weight gain as a consequence of exposure to different variables, such as specific diets.

On the other hand, we consider that the sample size used is one of the weak aspects in our study, pointing to the necessity to include new schools to increase the number of children. Nevertheless, from this first phase of the study, we expect to calculate the sample size needed to increase the statistical power and, consequently, to find solid associations between the studied variables. Furthermore, the classification of schools according to the area and the socioeconomic level widens the scope of the research, making it more representative of the city of Madrid. Similarly, the use of dietary and physical activity questionnaires may lead to reporting bias, but in the absence of better tools with low cost and high throughput, these records can offer valuable information, although it should be interpreted with caution.

After 5 years of follow-up, the GENYAL study aims to validate the machine learning predictive model that considers environmental and genetic factors in the obesity development, as well as the educational tools, to obtain new and potentially valuable data to increase our knowledge of the precipitants of childhood obesity and their relative importance to design preventive protocols at an early age based on machine learning models. With a view to the future perspective of continuity of this study, in addition to increasing the sample size to validate the results obtained, the possibility of implementing personalized nutritional education interventions is proposed to improve adherence and efficacy by applying the novel concepts provided by studies on precision nutrition.

## Data Availability Statement

The original contributions presented in the study are included in the article/supplementary materials, further inquiries can be directed to the corresponding author/s.

## Ethics Statement

The studies involving human participants were reviewed and approved by Ethics Committee of Fundación IMDEA-Food. Written informed consent to participate in this study was provided by the participants' legal guardian/next of kin.

## Author Contributions

VL-K was the principal investigator and responsible for the study and protocol design. HM-P helped designing the protocol and drafting the manuscript. HM-P, EA-A, RI, and IE-S were responsible for data collection. GC conducted statistical analysis of the data. SM contributed to genetic samples management. JM, GR, and AR supervised the final compilation of the manuscript and provided scientific advice and consultation. All authors read and approved the final manuscript.

## Funding

This study was supported by Conserjería de Educación, Universidades y Ciencia de la Comunidad de Madrid, Dirección General de Educación Infantil, Primaria y Secundaria.

## Conflict of Interest

The authors declare that the research was conducted in the absence of any commercial or financial relationships that could be construed as a potential conflict of interest.

## Publisher's Note

All claims expressed in this article are solely those of the authors and do not necessarily represent those of their affiliated organizations, or those of the publisher, the editors and the reviewers. Any product that may be evaluated in this article, or claim that may be made by its manufacturer, is not guaranteed or endorsed by the publisher.

## Abbreviations

ACM: Autonomous Community of Madrid
SNPs: Single Nucleotide Polymorphisms
IPAC: Individual Physical Activity Coefficient
PAC: Physical Activity Coefficient
EW: Excess Weight.
